# Supplementary material for: Construction of 3D copper-chitosan-gas diffusion layer electrode for highly efficient CO2 electrolysis to C2+ alcohols
Source: Nat Commun. 2023 May 17;14:2823. doi: 10.1038/s41467-023-38524-3 (PMC10192345; doi:10.1038/s41467-023-38524-3)
Supplement: Supplementary file 1 — Supplementary Information [file 41467_2023_38524_MOESM1_ESM.pdf]

Supplementary Information

Construction of 3D copper-chitosan-gas diffusion layer electrode for highly efficient CO_2 electrolysis to C_{2+} alcohols

Jiahui Bi[1,2], Pengsong Li[1,2], Jiyuan Liu[1,2], Shuaiqiang Jia[3], Yong Wang[1,2], Qinggong Zhu[1,2*], Zhimin Liu[1,2], and Buxing Han[1,2,3*]

[1]Beijing National Laboratory for Molecular Sciences, CAS Key Laboratory of Colloid, Interface and Chemical Thermodynamics, CAS Research/Education Center for Excellence in Molecular Sciences, Center for Carbon Neutral Chemistry, Institute of Chemistry, Chinese Academy of Sciences, Beijing 100190, P. R. China

[2]School of Chemistry and Chemical Engineering, University of Chinese Academy of Sciences, Beijing 100049, P. R. China

[3]Shanghai Key Laboratory of Green Chemistry and Chemical Processes, School of Chemistry and Molecular Engineering, East China Normal University Shanghai 200062, P. R. China

*E-mail: qgzhu@iccas.ac.cn; hanbx@iccas.ac.cn

Supplementary Figures (Pages S1-S38)

Supplementary Tables (Pages S39-S40)

Supplementary References (Pages S41-S42)

Supplementary Figures

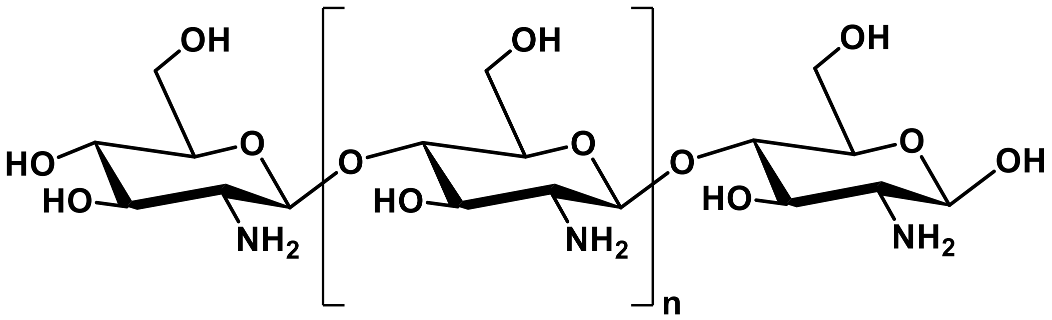

Figure S1. The structural formula of CS.

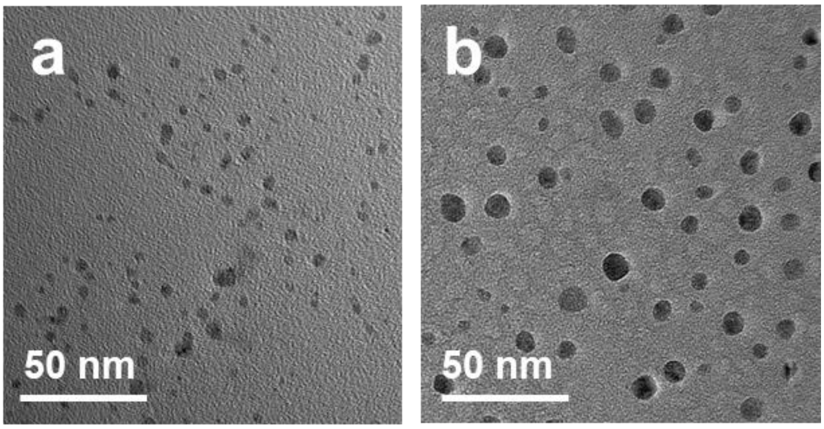

Figure S2. TEM images of Cu NPs in a) pre-Cu-CS-1 and b) pre-Cu-CS-2.

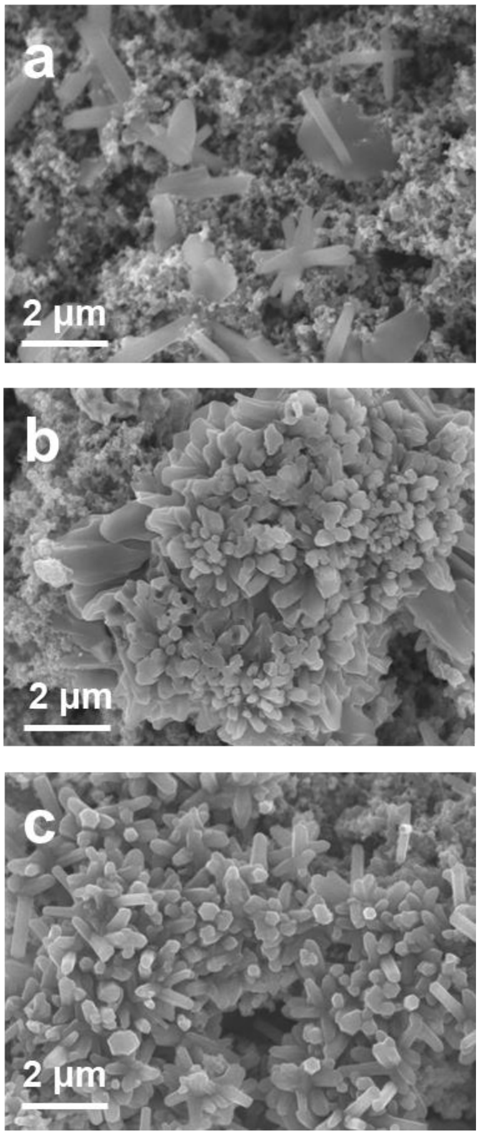

Figure S3. SEM images of 3D Cu-CS-GDL at different time of in situ reconstruction step. a) 3D Cu-CS-GDL-1 min; b) 3D Cu-CS-GDL-5min; c) 3D Cu-CS-GDL-10 min.

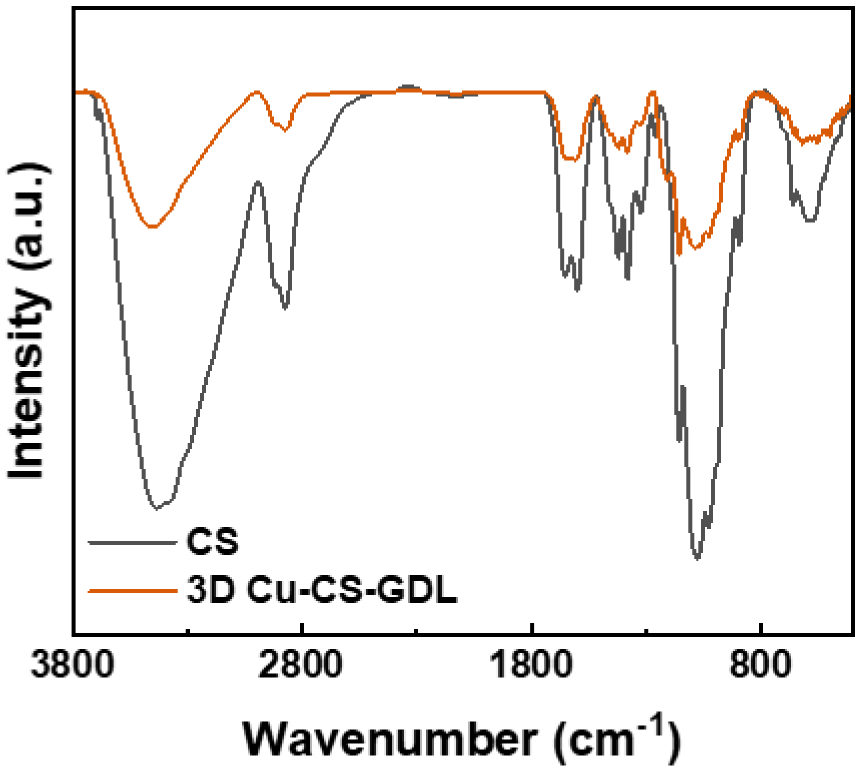

Figure S4. The FT-IR data of 3D Cu-CS-GDL and CS.

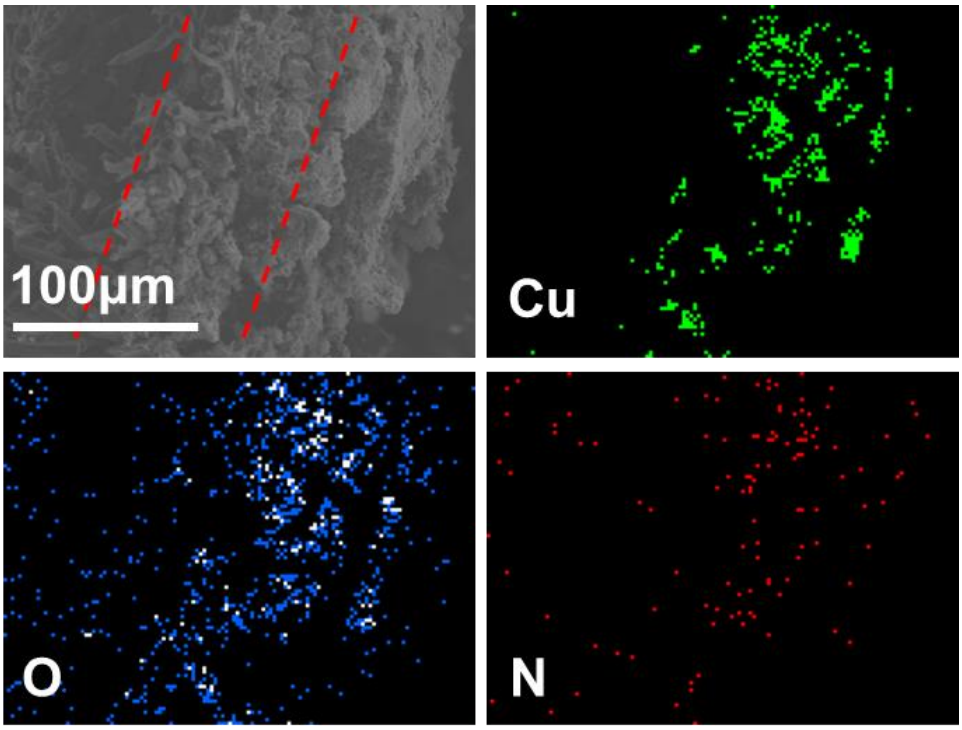

Figure S5. The side view EDS of 3D Cu-CS-GDL.

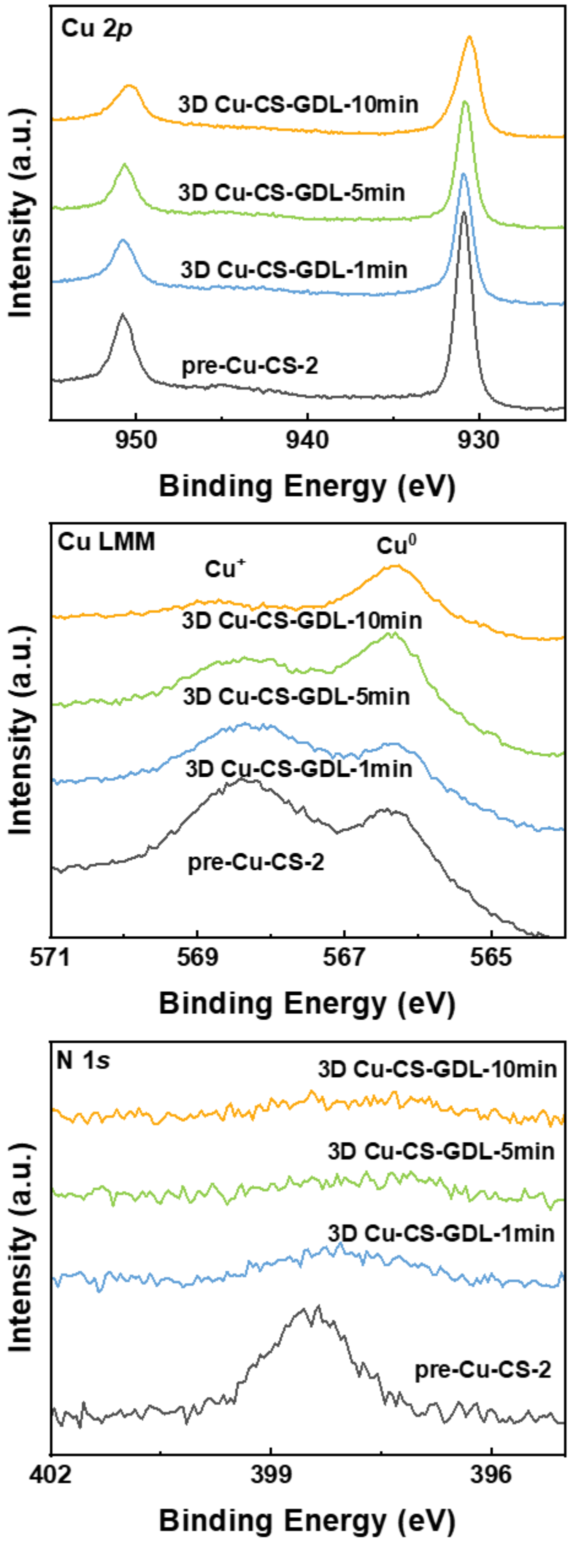

Figure S6. Semi in-situ XPS spectra of Cu 2*p*, Cu LMM Auger and N 1*s* orbits of pre-Cu-CS-2 and 3D Cu-CS-GDL.

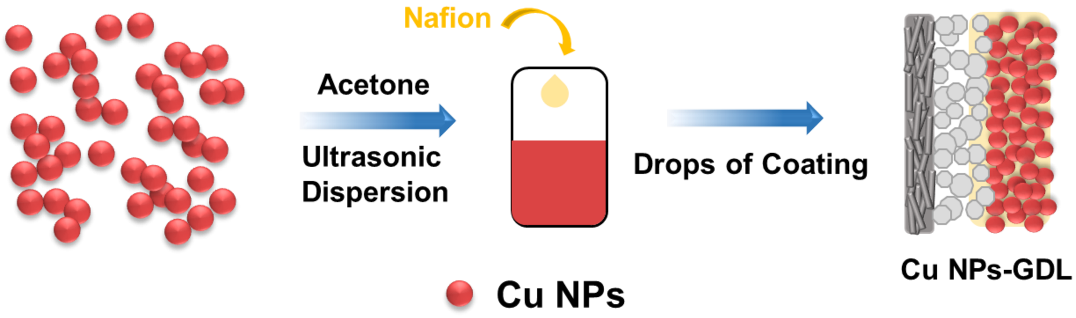

Figure S7. Schematic diagram of the preparation of Cu NPs-GDL electrode.

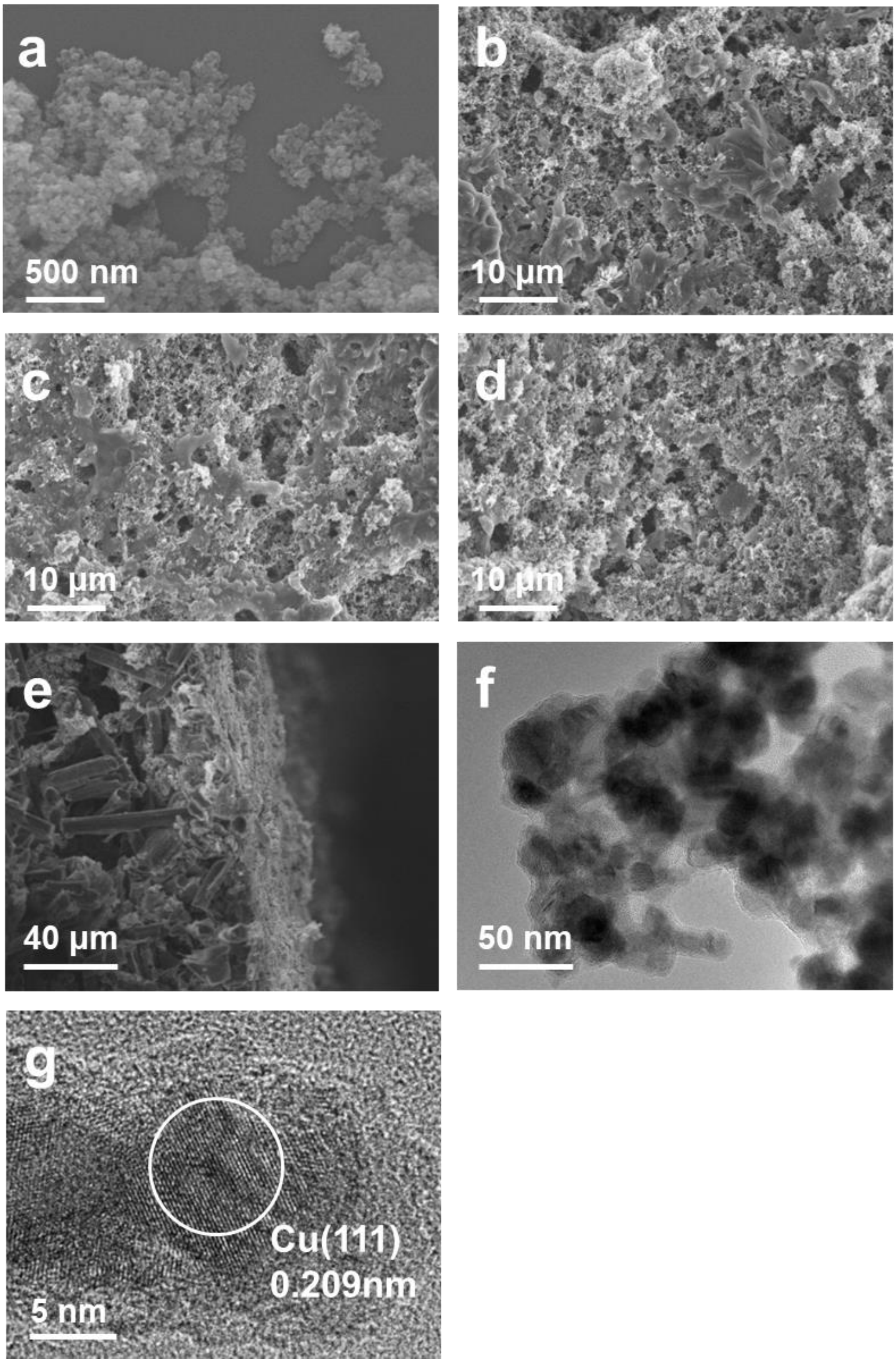

Figure S8. The SEM images of a) the as-synthesized Cu NPs before CO_2RR, b) Cu NPs-GDL after 1 min of CO_2RR, c) Cu NPs-GDL after 5 min of CO_2RR and d) Cu NPs-GDL after 10 min of CO_2RR. e) The SEM of the side view of Cu NPs-GDL-10 min; f) the TEM image of Cu NPs-GDL-10 min; g) the HRTEM image of Cu NPs-GDL-10 min.

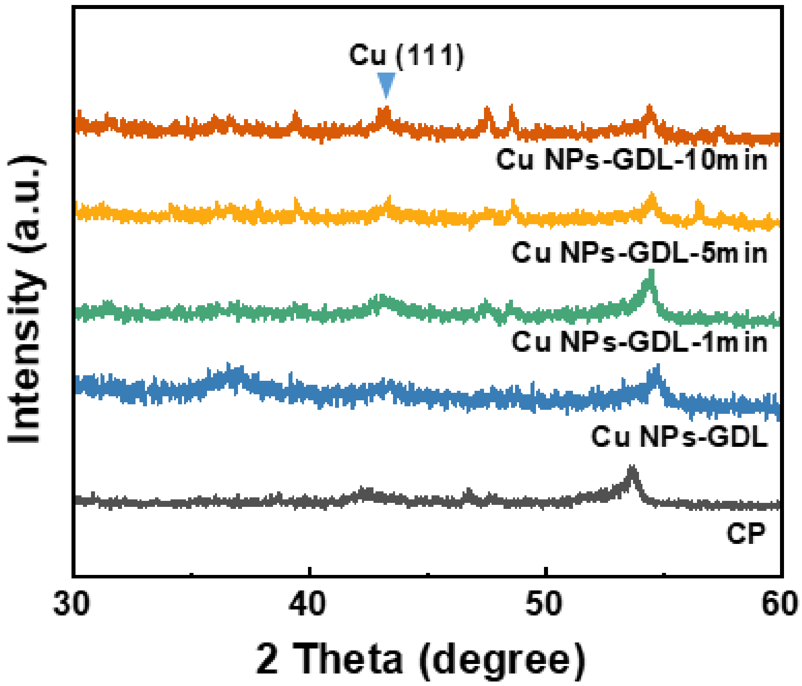

Figure S9. XRD patterns of CP, Cu NPs-GDL before and after different time of CO_2RR.

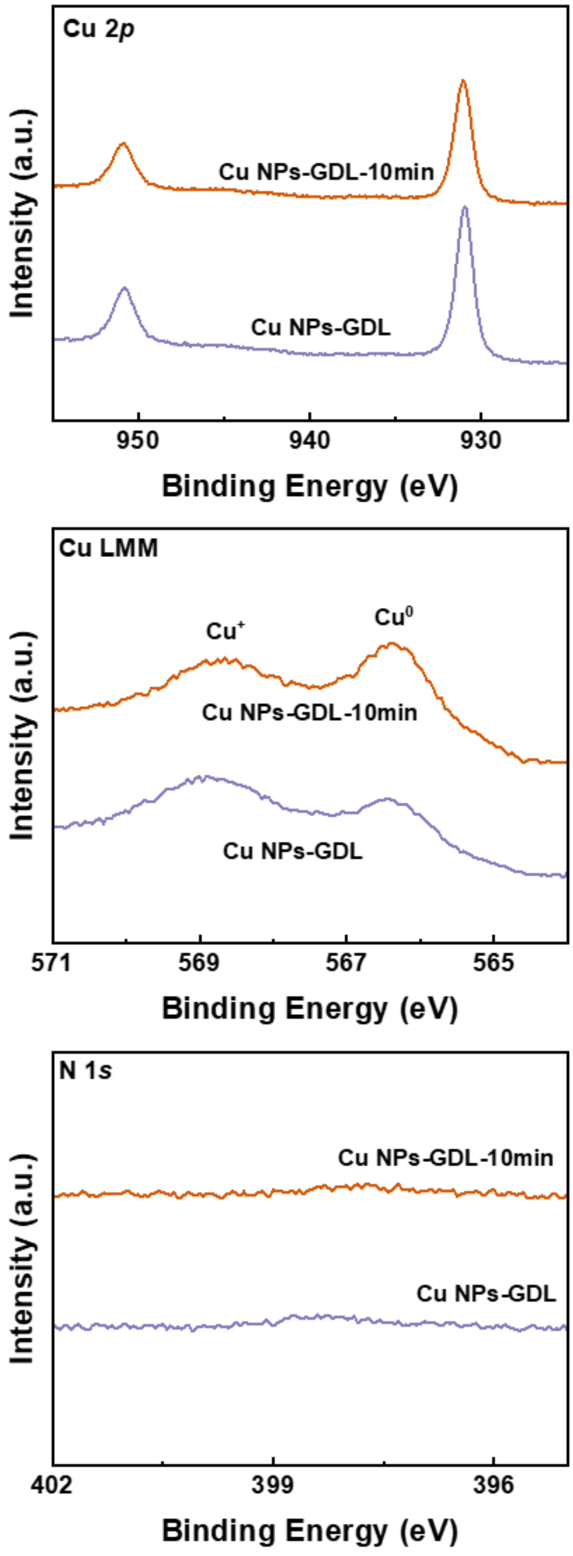

Figure S10. Semi in-situ XPS spectra of Cu 2*p*, Cu LMM Auger and N 1*s* orbits of Cu NPs-GDL before and after 10 min of CO_2RR.

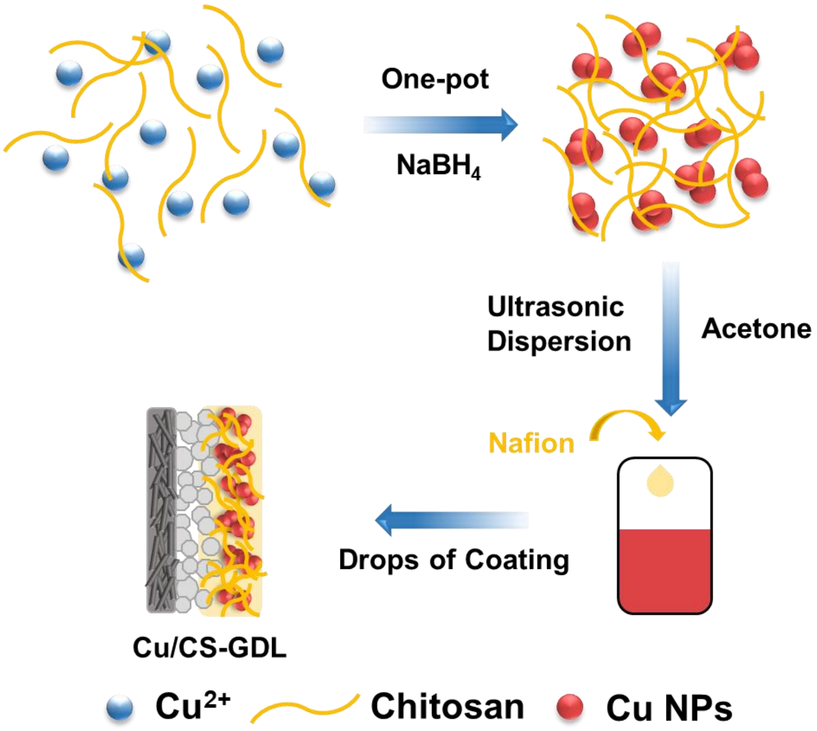

Figure S11. Schematic diagram of the preparation of Cu/CS-GDL electrode.

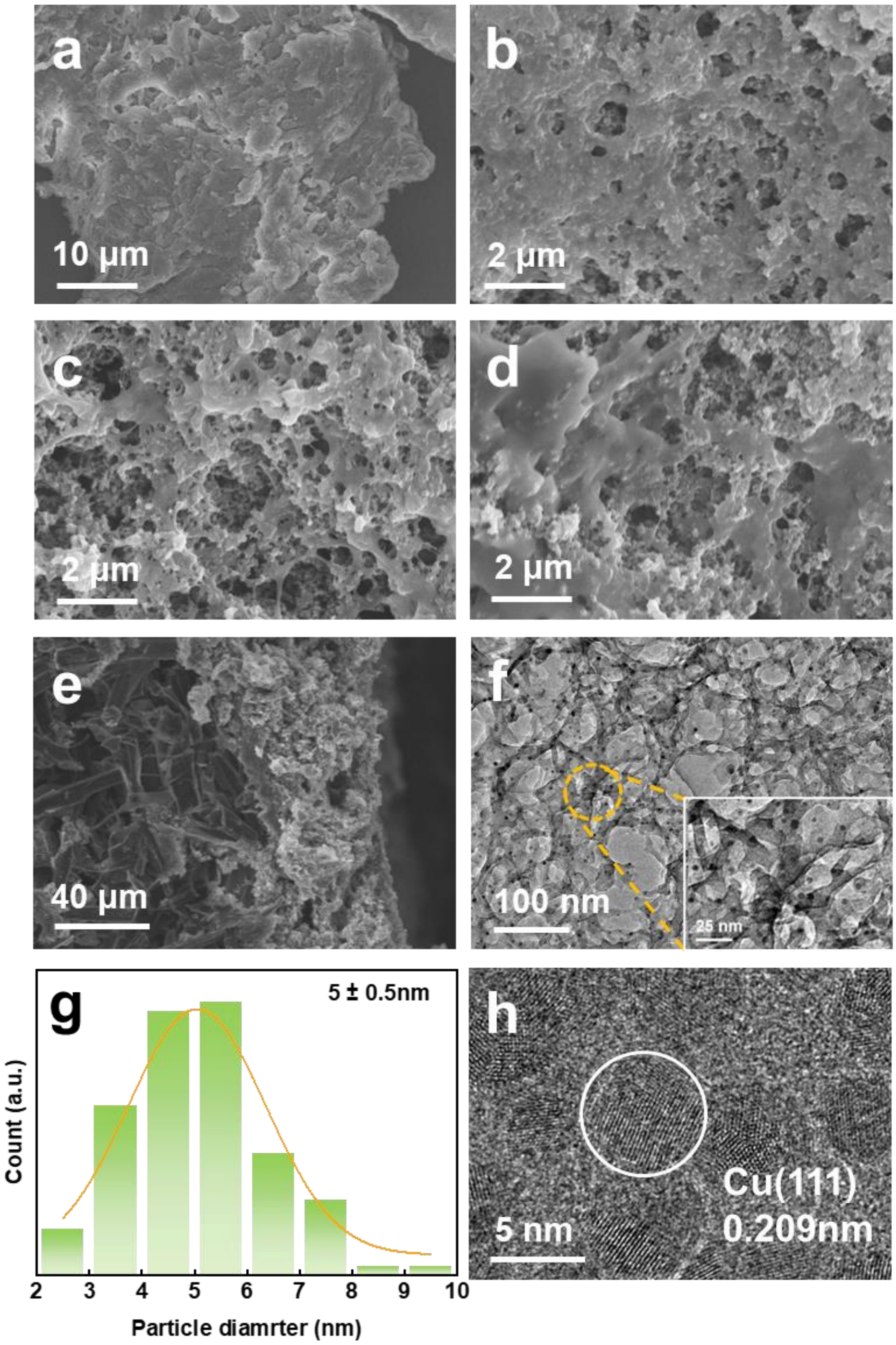

Figure S12. The SEM images of a) the as-synthesized Cu/CS composite before CO_2RR, b) Cu/CS-GDL after 1 min of CO_2RR, c) Cu/CS-GDL after 5 min of CO_2RR and d) Cu/CS-GDL after 10 min of CO_2RR. e) The SEM of the side view of Cu/CS-GDL-10 min; f) the TEM images of Cu/CS-GDL-10min (inset: high-magnification); g) The particle size distribution of Cu NPs in Cu/CS-GDL-10min; h) the HRTEM image of Cu/CS-GDL-10min.

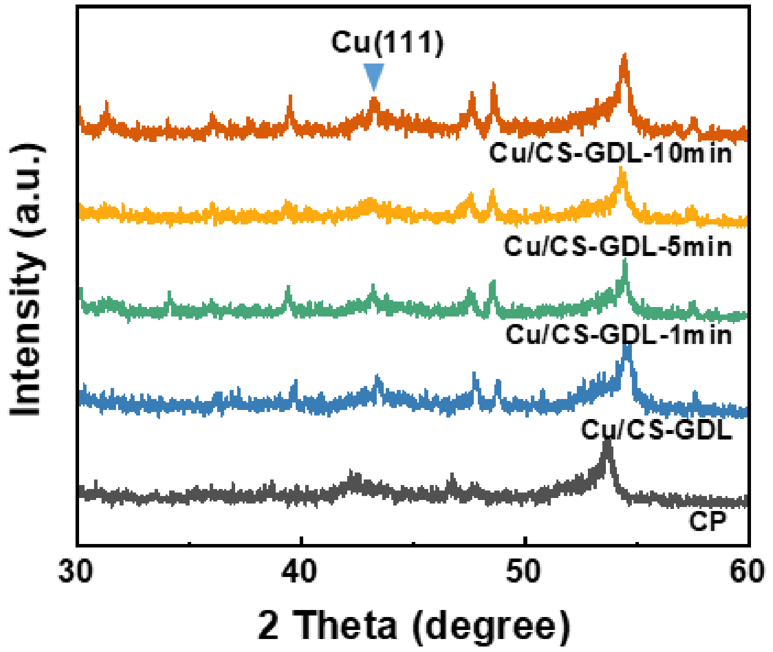

Figure S13. XRD patterns of CP, Cu/CS-GDL before and after different time of CO_2RR.

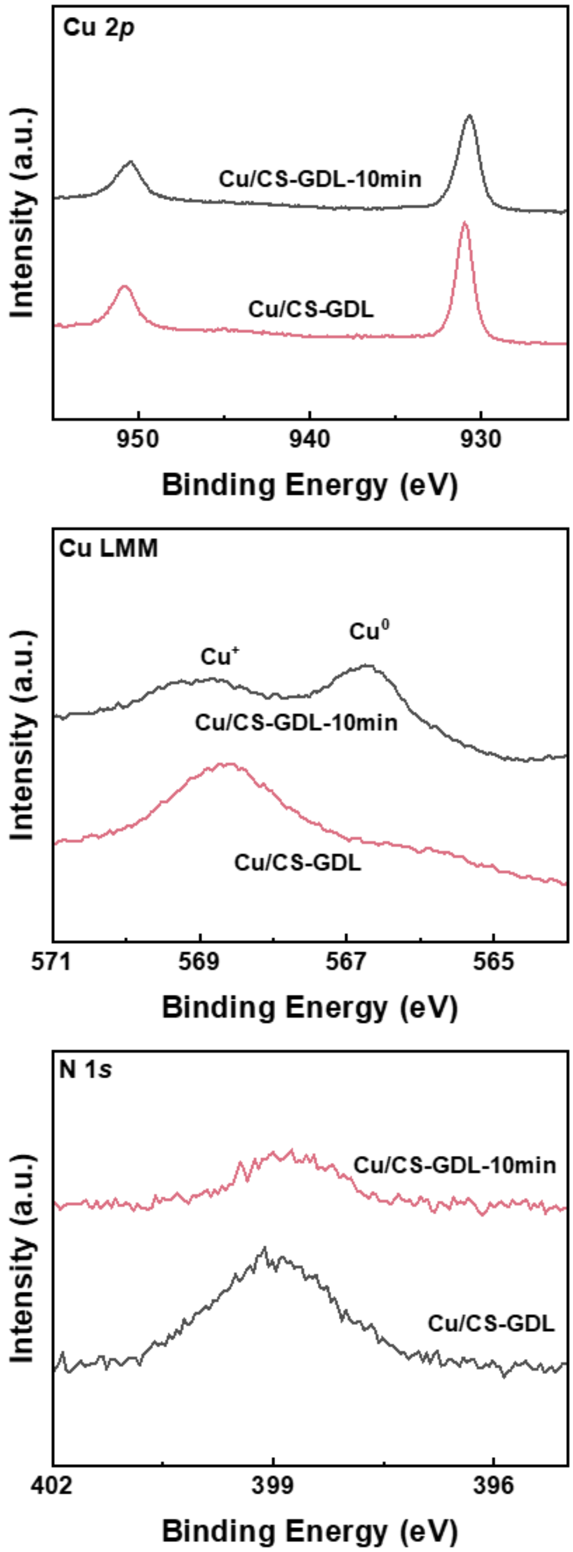

Figure S14. Semi in-situ XPS spectra of Cu 2*p*, Cu LMM Auger and N 1*s* orbits of Cu/CS-GDL before and after 10 min of CO_2RR.

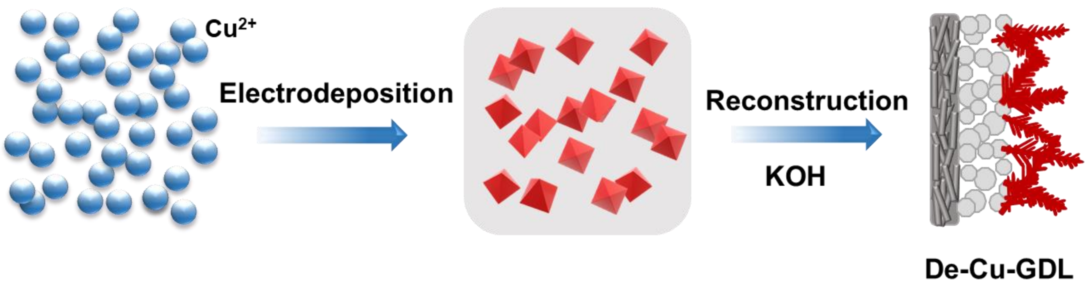

Figure S15. Schematic diagram of the preparation of De-Cu-GDL electrode.

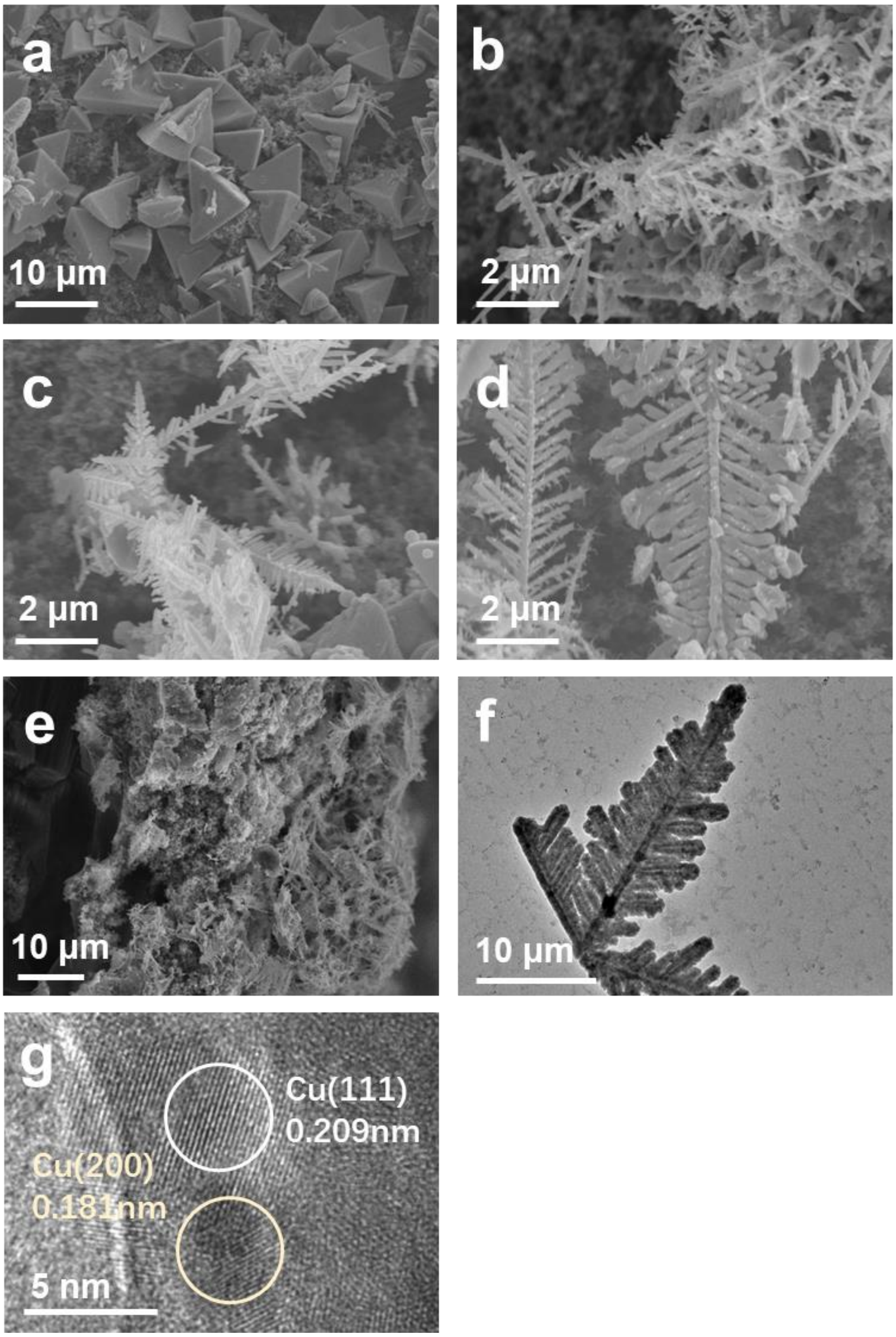

Figure S16. The SEM images of a) pre-De-Cu catalyst, b) De-Cu-GDL after 1 min of CO_2RR, c) De-Cu-GDL after 5 min of CO_2RR and d) De-Cu-GDL after 10 min of CO_2RR. e) The SEM of the side view of De-Cu-GDL-10 min; f) the TEM image of De-Cu-GDL-10 min; g) the HRTEM image of De-Cu-GDL-10 min.

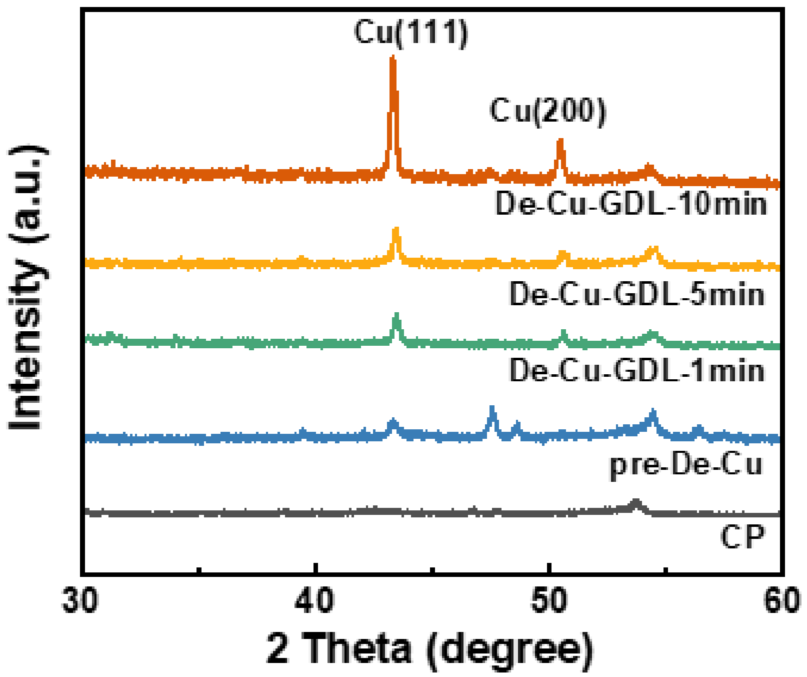

Figure S17. XRD patterns of CP, pre-De-Cu and De-Cu-GDL after different time of CO_2RR.

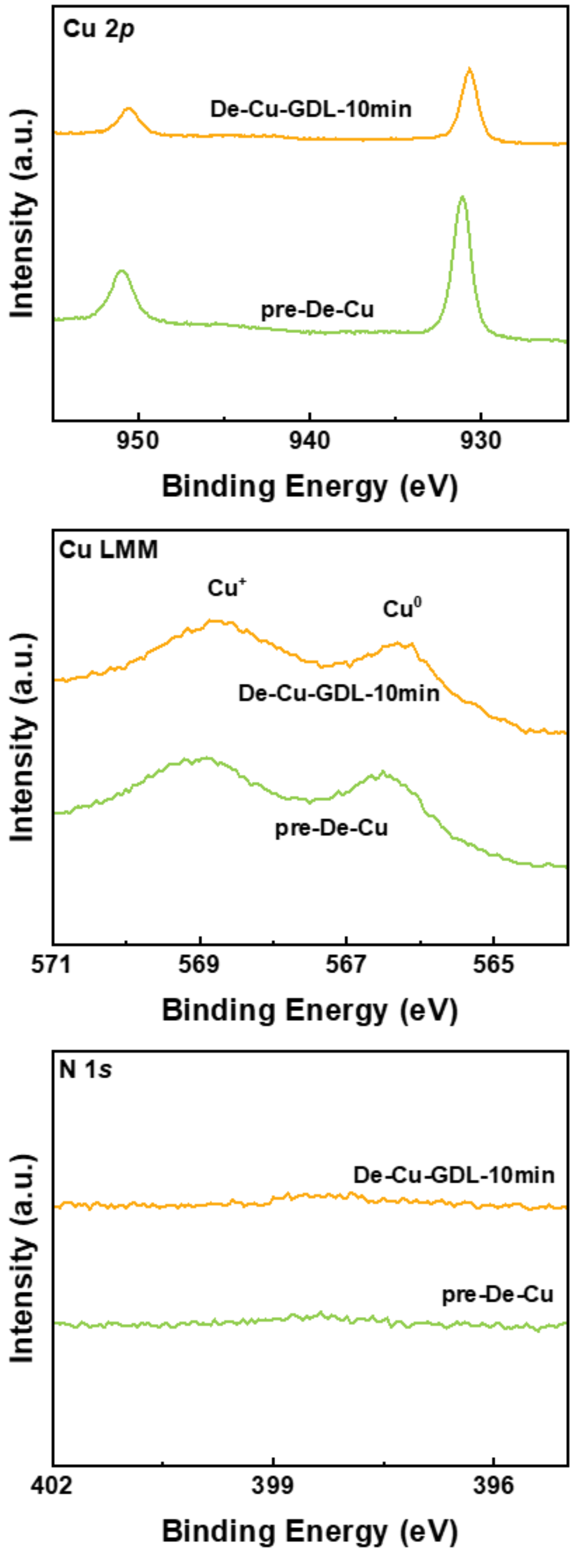

Figure S18. Semi in-situ XPS spectra of Cu 2*p*, Cu LMM Auger and N 1*s* orbits of pre-De-Cu and De-Cu-GDL after 10 min of CO_2RR.

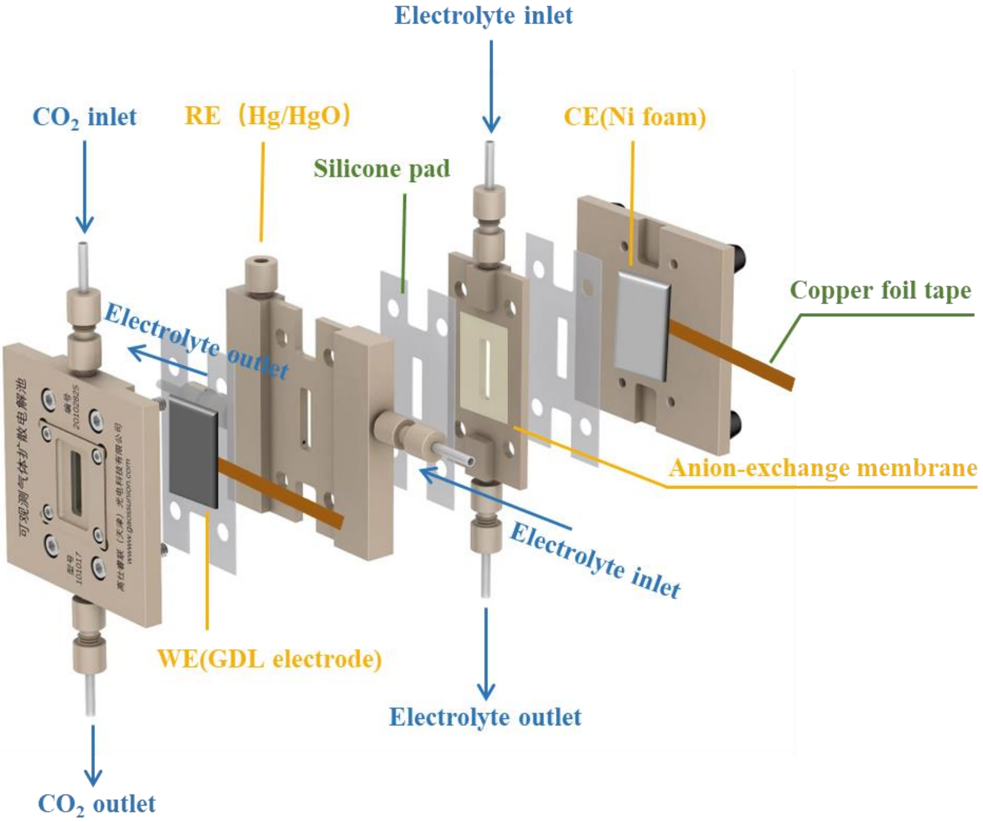

Figure S19. Diagram of a flow cell. WE, RE and CE stand for working electrode, reference electrode and counter electrode, respectively.

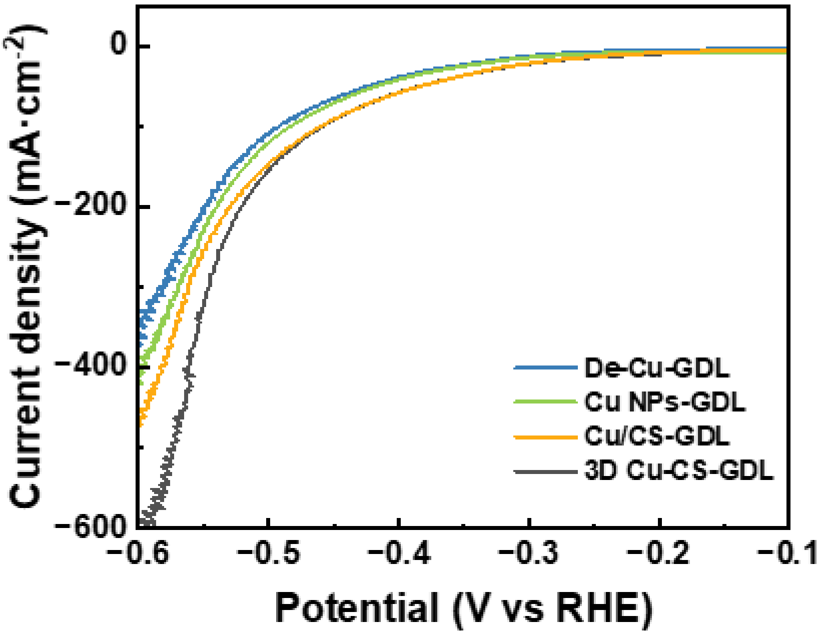

Figure S20. Linear sweep voltammetry (LSV) curves of various GDEs with the scan rate of 20 mV·s^{-1}.

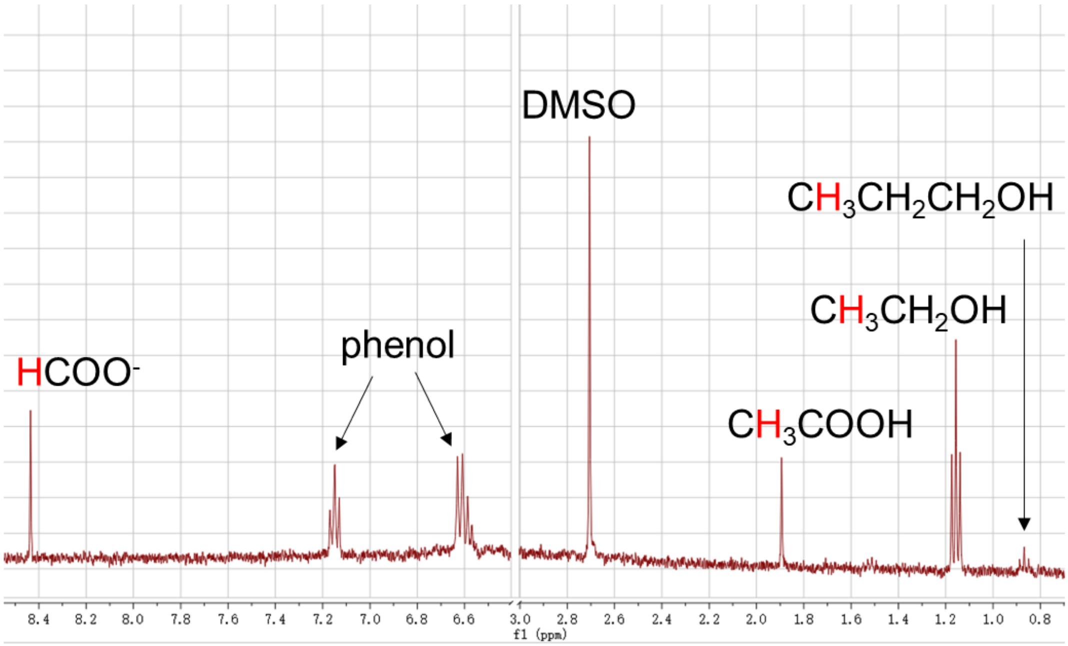

Figure S21. Typical ^{1}H NMR spectra of freshly acquired liquid samples of 3D Cu-CS-GDL electrode after CO_2RR.

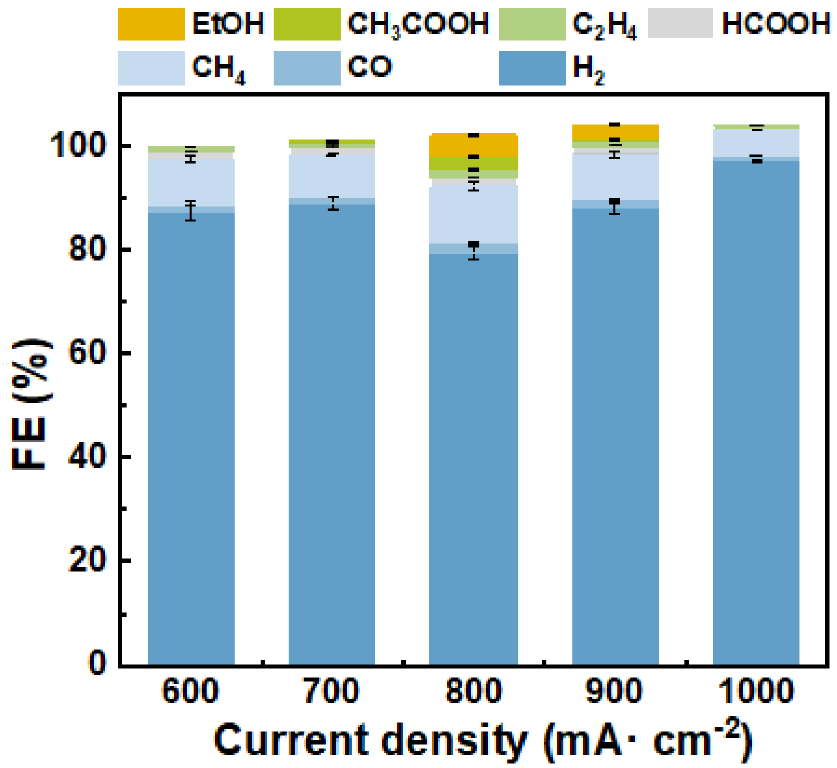

Figure S22. The product distribution of CO_2RR on Cu/CS-GDL electrode ranging from 600 to 1000 mA·cm^{-2}. Error bars denote the standard deviations from multiple measurements.

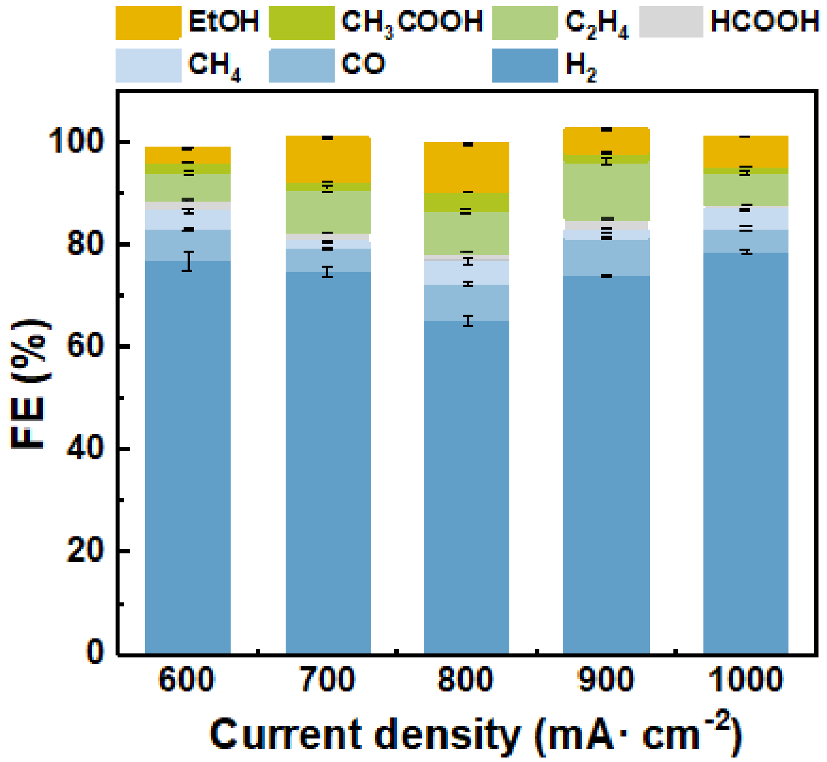

Figure S23. The product distribution of CO_2RR on De-Cu-GDL electrode ranging from 600 to 1000 mA·cm^{-2}. Error bars denote the standard deviations from multiple measurements.

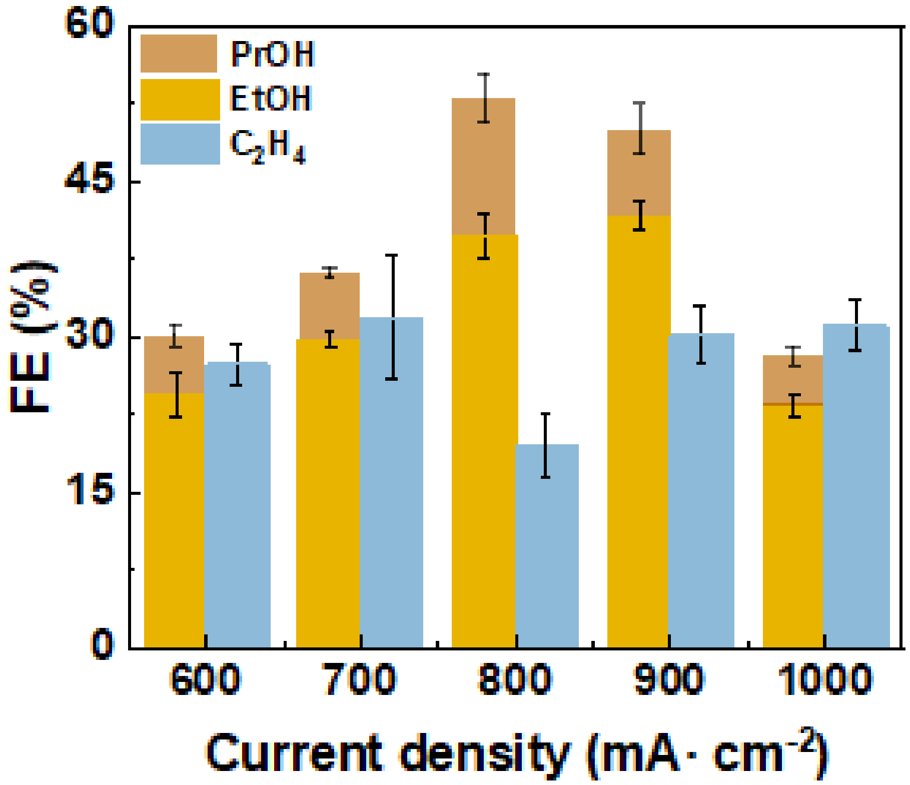

Figure S24. C_{2+} alcohols and C_2H_4 FE values on 3D Cu-CS-GDL ranging from 600 to 1000 mA · cm^{-2}. Error bars denote the standard deviations from multiple measurements.

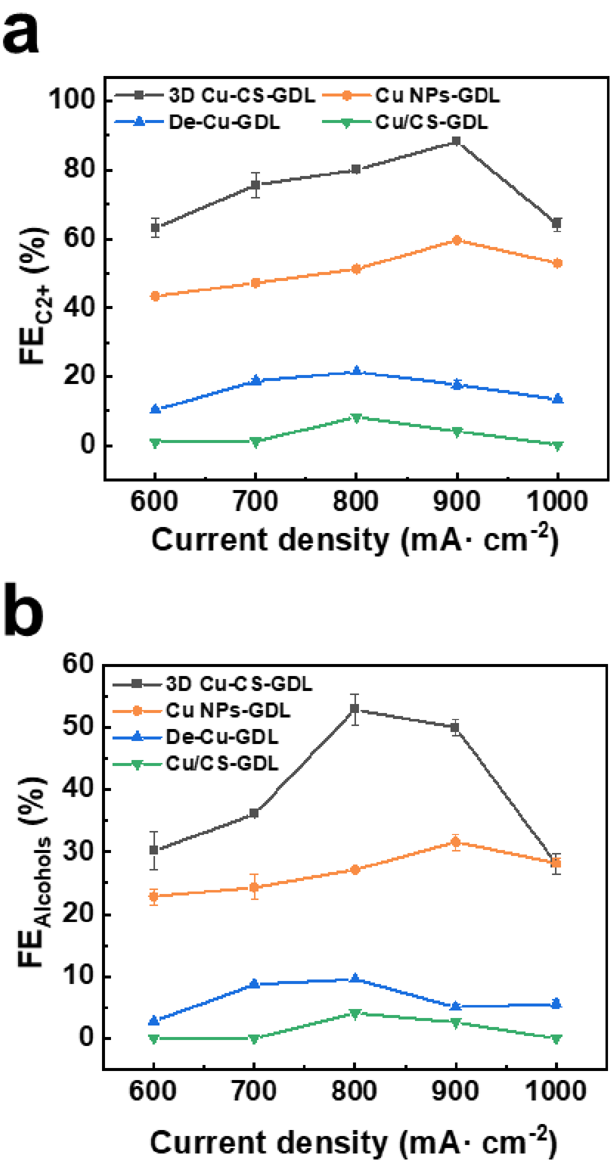

Figure S25. The FE of a) C_{2+} products and b) C_{2+} alcohols on different GDEs at various current densities ranging from 600 to 1000 mA·cm^{-2}. Error bars denote the standard deviations from multiple measurements.

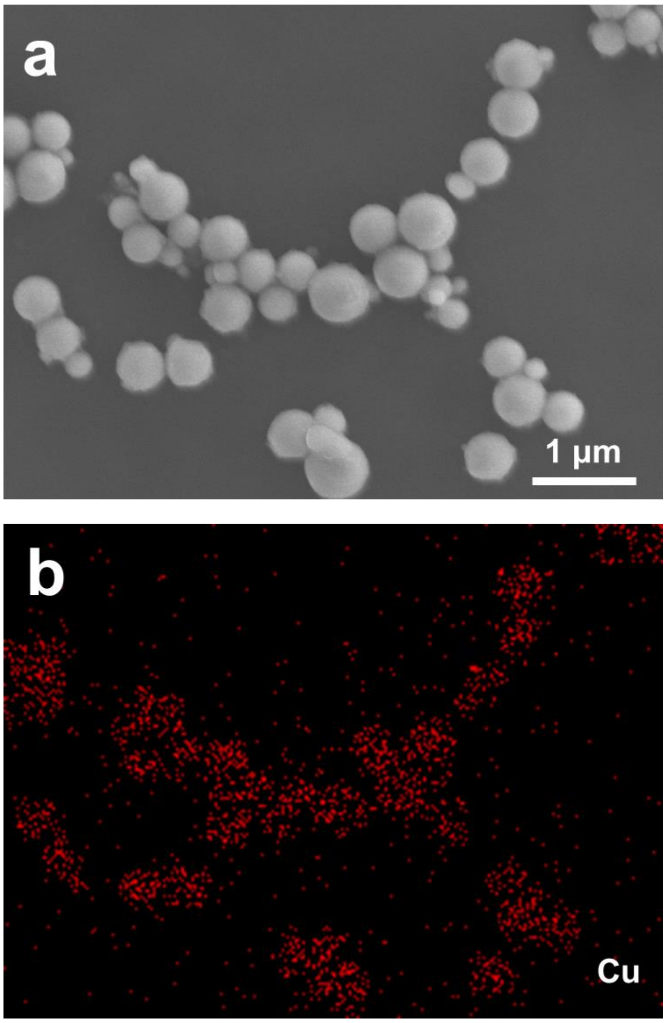

Figure S26. a) The SEM image and b) the EDS image of commercial Cu NPs.

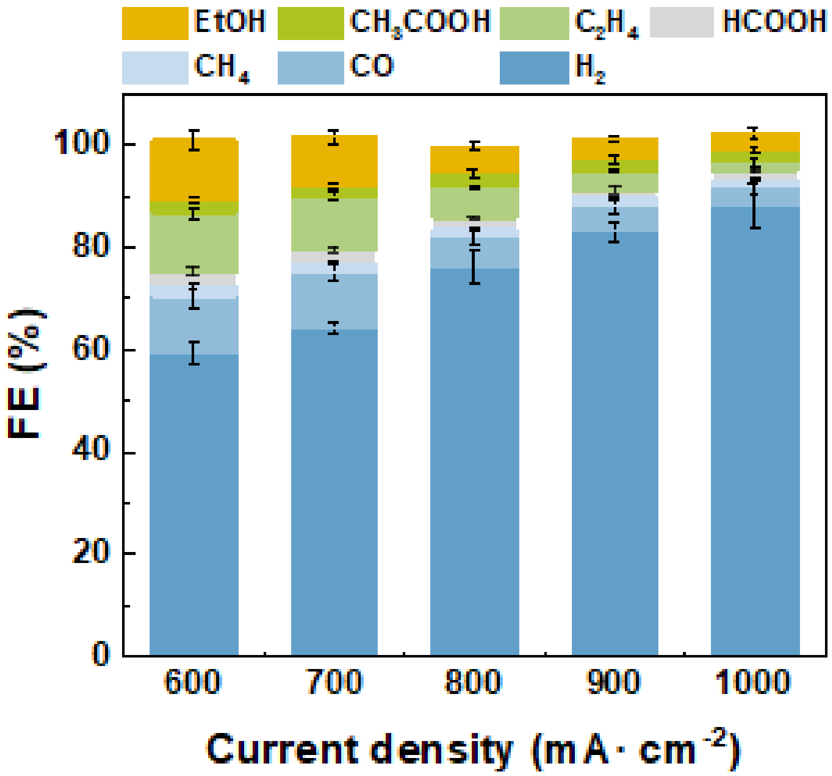

Figure S27. Product distributions of CO_2RR on commercial Cu NPs. Error bars denote the standard deviations from multiple measurements.

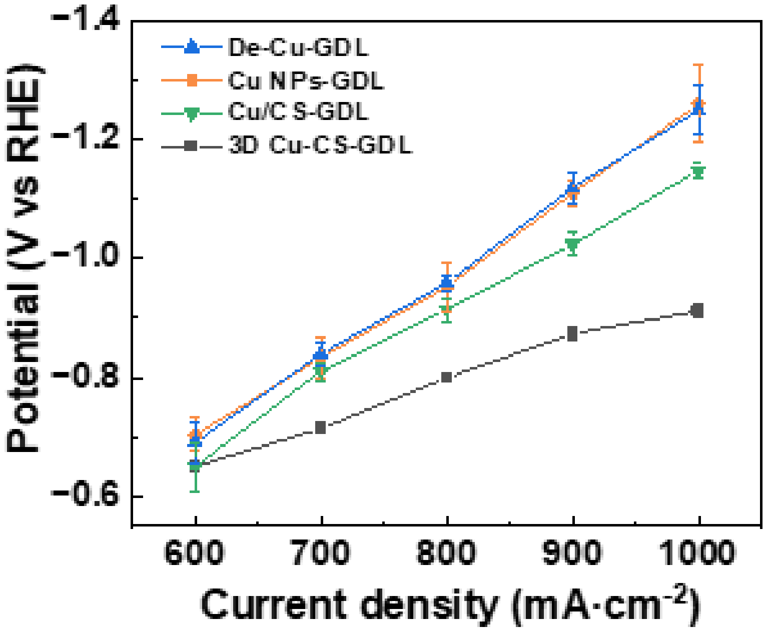

Figure S28. Total current densities vs applied potentials over different GDEs. Error bars represent the standard deviation of potentials under constant-current mode.

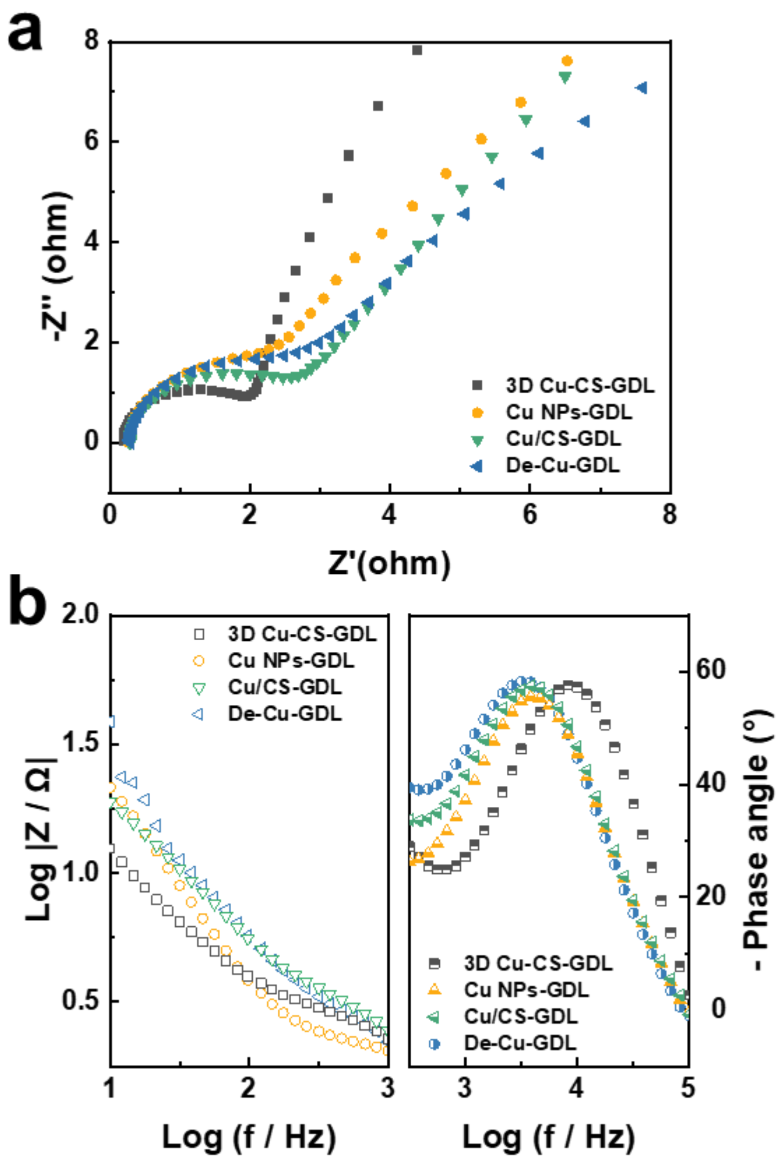

Figure S29. a) Nyquist and b) Bode plots of various electrodes in 1M KOH in CO_2 atmosphere at open circuit voltage in a flow cell.

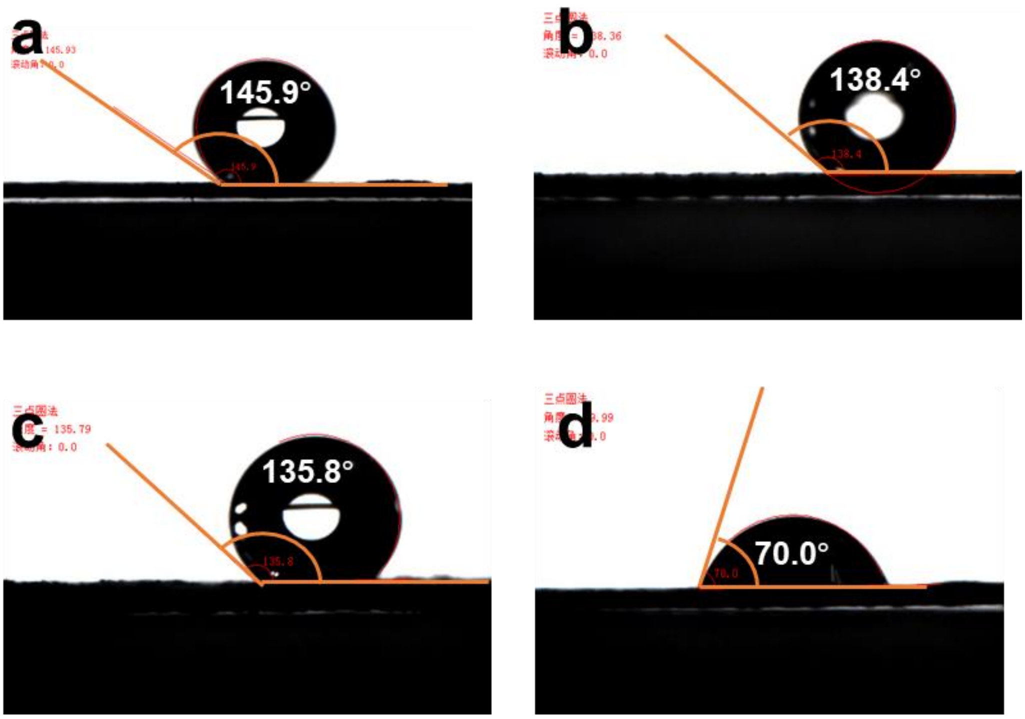

Figure S30. The contact angle data of a) hydrophobic CP, b) Cu NPs-GDL, c) Cu/CS-GDL and d) De-Cu-GDL, respectively.

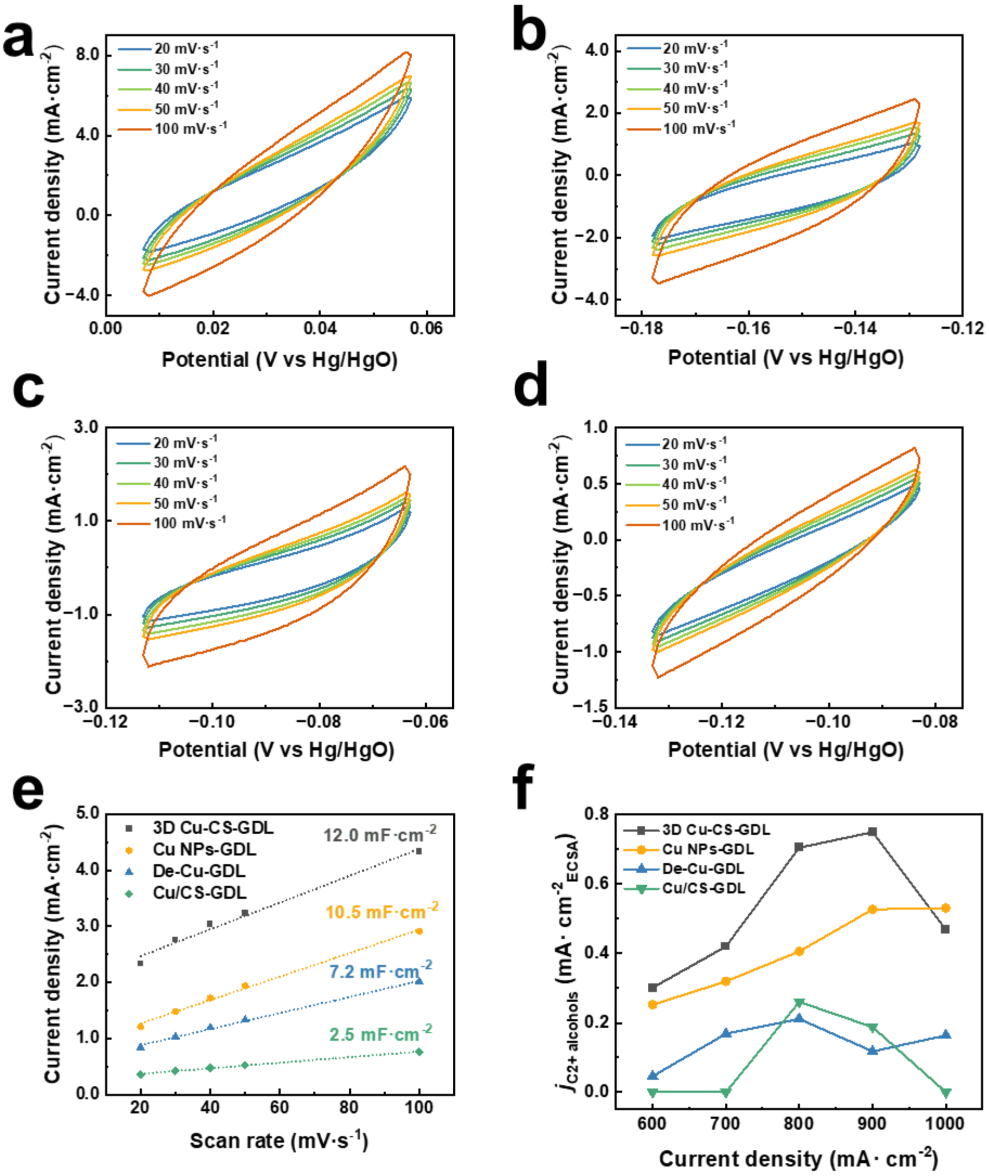

Figure S31. Electric double layer capacitance (C_{dl}) measurements at the non-Faradaic region with various scan rates (20 mV·s^{-1}- 100 mV·s^{-1}) of a) 3D Cu-CS-GDL electrode (from 0.057 to 0.007 V vs Hg/HgO), b) Cu NPs-GDL electrode (from -0.128 to -0.178 V vs Hg/HgO), c) De-Cu-GDL electrode (from -0.063 to -0.113 V vs Hg/HgO) and d) Cu/CS-GDL electrode (from -0.083 to -0.133 V vs Hg/HgO. e) Charging current density differences plotted against scan rates. f) The ESCA-normalized partial current densities of C_{2+} alcohols of GDEs at 600-1000 mA·cm^{-2}.

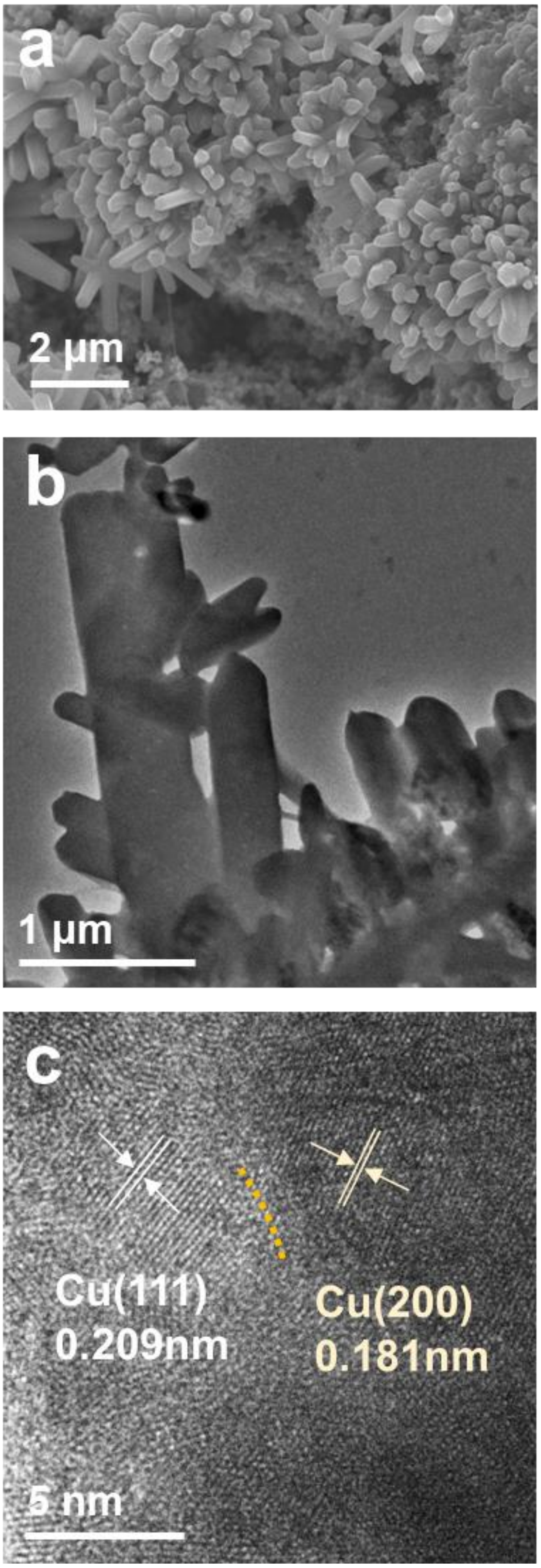

Figure S32. a) The SEM, b) TEM and c) HRTEM images of 3D Cu-CS-GDL after CO_2RR.

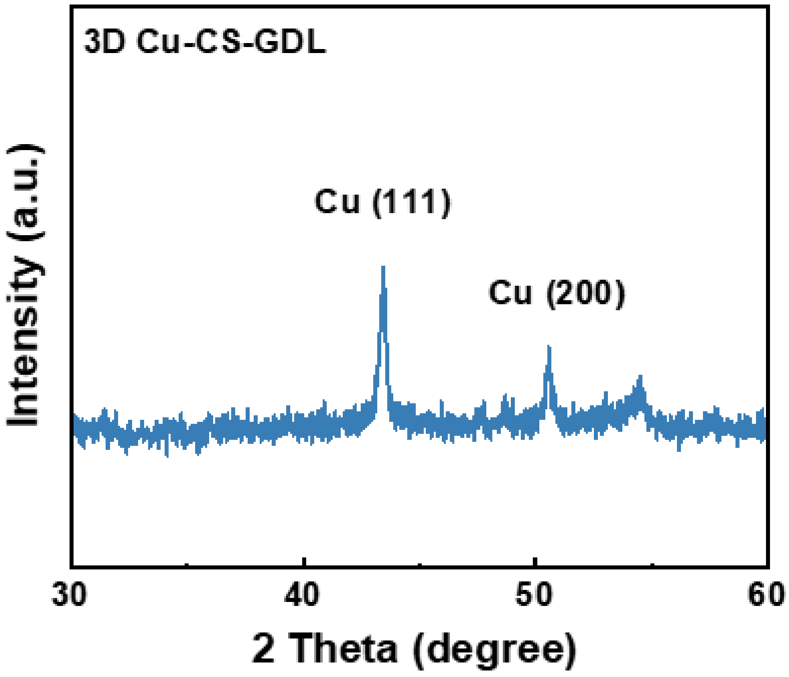

Figure S33. XRD pattern of 3D Cu-CS-GDL after CO_2RR.

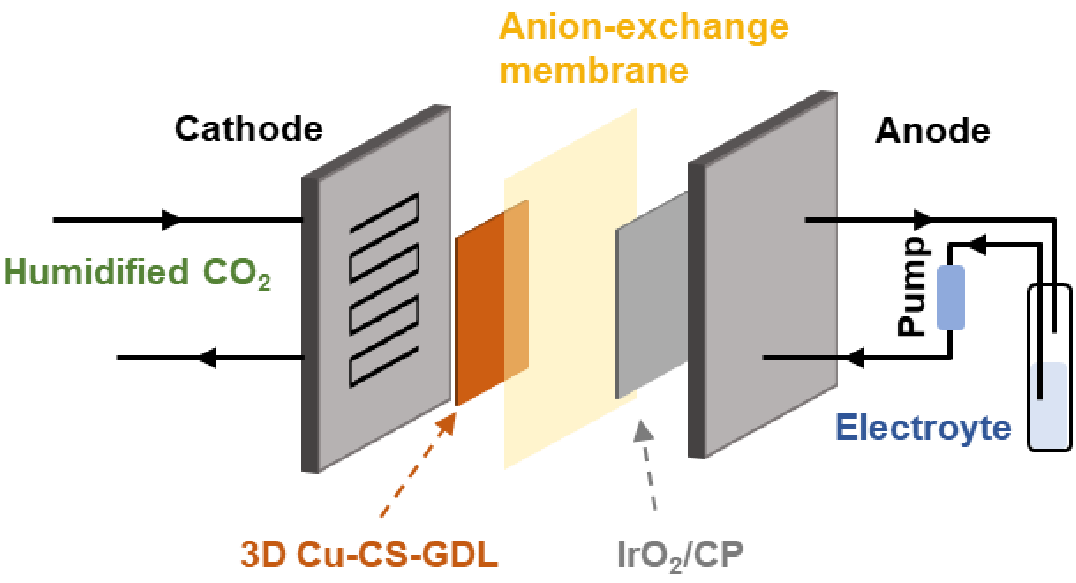

Figure S34. The schematic diagram of MEA.

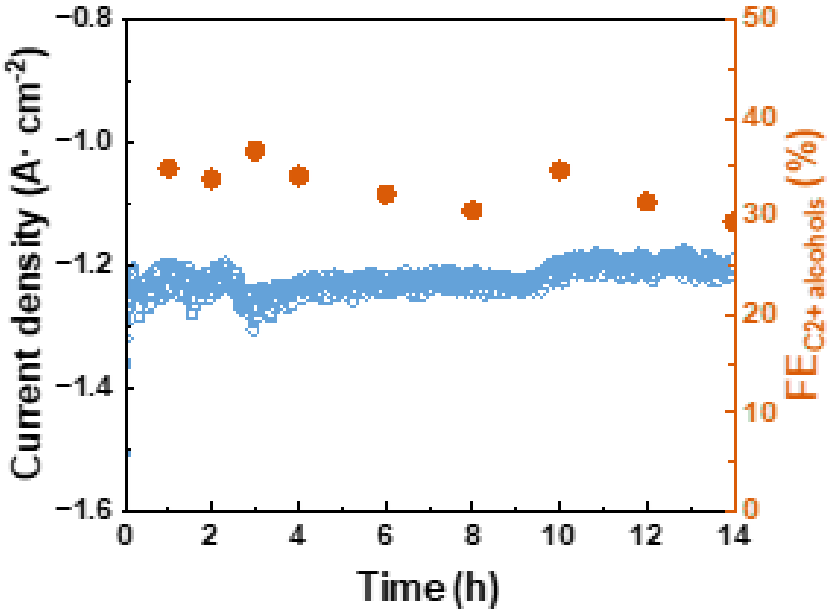

Figure S35. The stability of 3D Cu-CS-GDL electrode at -3.6 V in MEA.

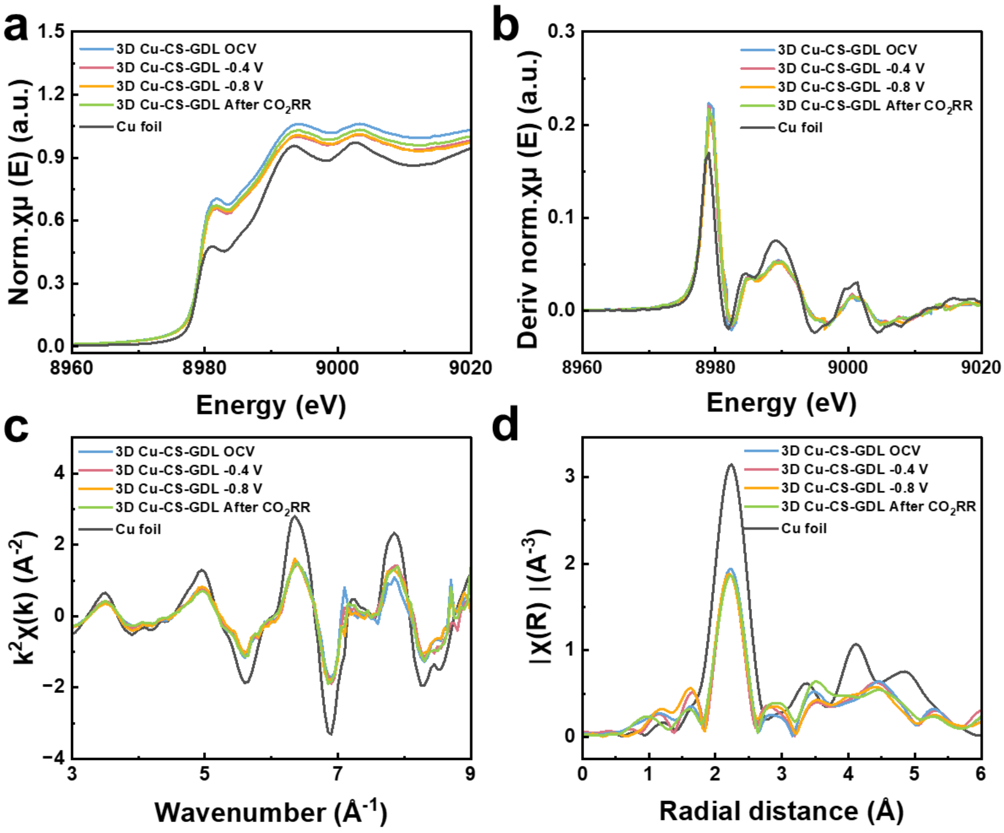

Figure S36. a) Cu K-edge XANES spectra; b) Cu derivative K-edge XANES spectra; c) Cu EXAFS spectra in k space; d) Cu K-edge FT of the EXAFS spectra. The XAFS data of 3D Cu-CS-GDL was obtained at various potentials (V vs RHE) during CO_2RR. The OCV means open-circuit voltage.

.

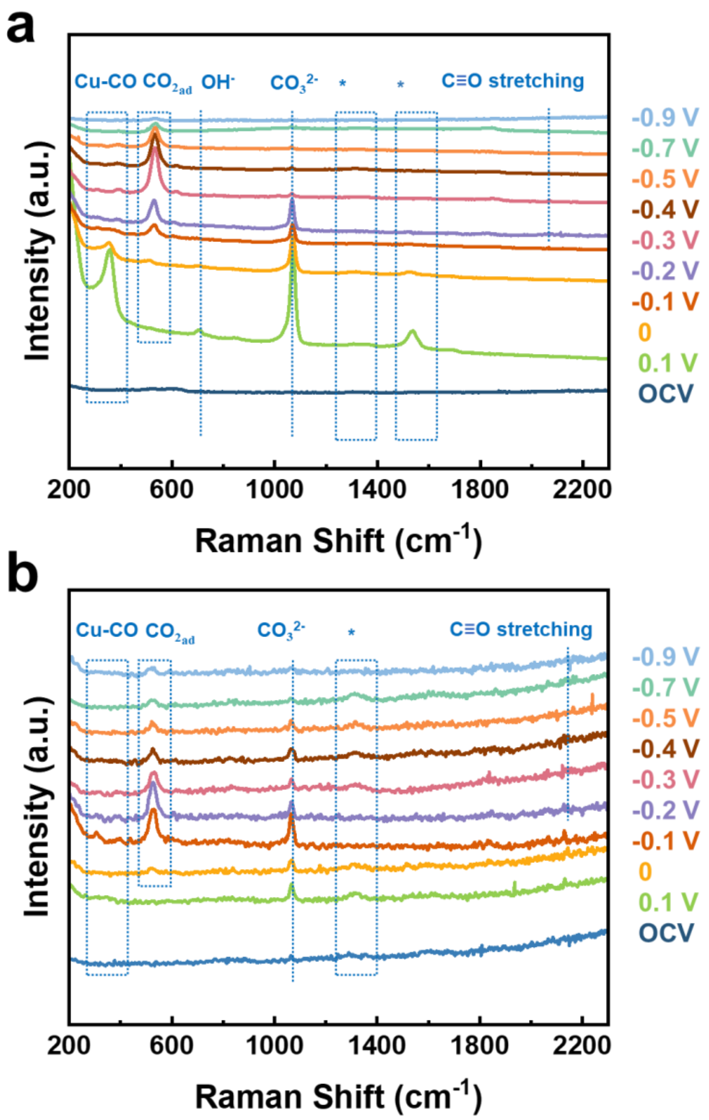

Figure S37. a) The in-situ Raman spectra for 3D Cu-CS-GDL electrode at various potentials (V vs RHE) during CO_2RR; b) The in-situ Raman spectra for Cu NPs-GDL electrode at various potentials (V vs RHE) during CO_2RR. The OCV means open-circuit voltage.

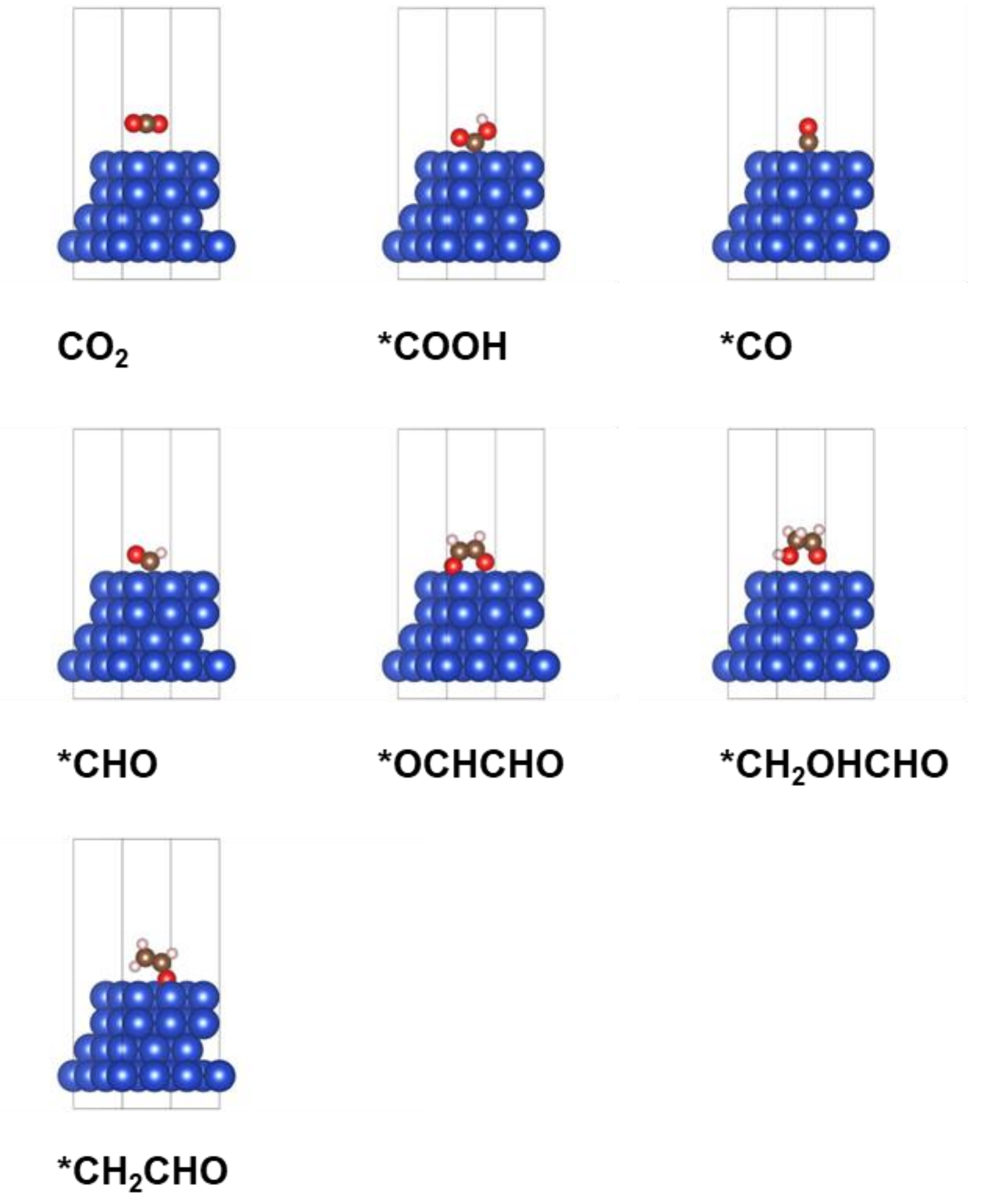

Figure S38. The adsorption models of CO_2 and intermediates on the Cu (111) crystal face.

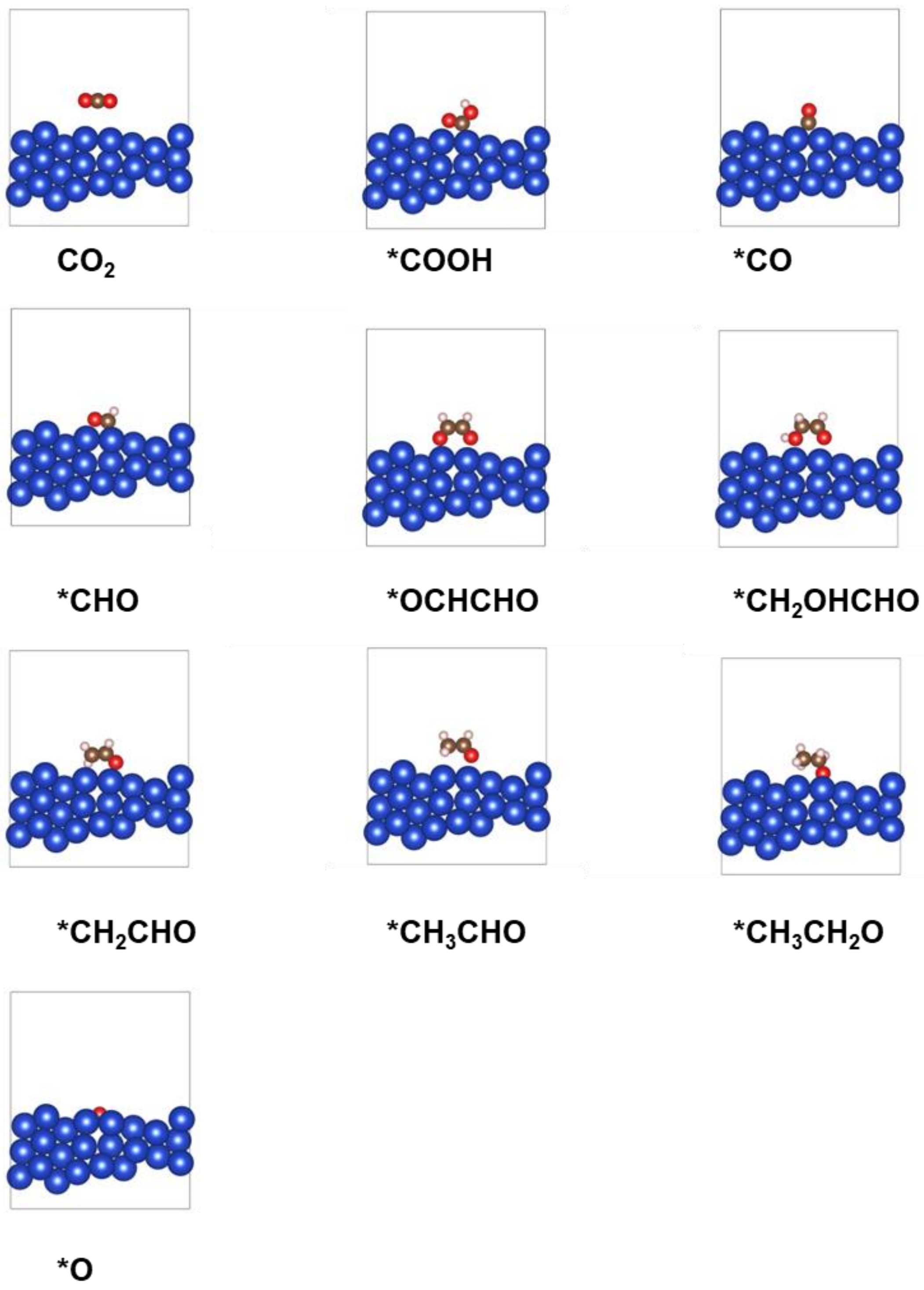

Figure S39. The adsorption models of CO_2 and intermediates on the heterojunction between Cu (111) and Cu (200) plane.

Supplementary Tables

Table S1. Comparison of FE_{C2+} and current density (*j*, geometric normalized) over 3D Cu-CS-GDL architecture with some typical Cu-based catalysts in CO_2RR.

Electrodes	Potential (V vs RHE)	j_{total} ($mA \cdot cm^{-2}$)	FE_{C2+} (%)	$FE_{alcohols}$ (%)	$j_{C2+\ alcohols}$ ($mA \cdot cm^{-2}$)	Ref.
3D Cu-CS-GDL	-0.87	900	88.2	51.4	462.6	This work
3D Cu-CS-GDL	-0.80	800	80.0	54.7	437.6	
Fluorine-modified Cu	-0.89	1600	80	13	208	1
AEI-OD-Cu	-0.78	800	85.1	15	120	2
Nanoporous Cu	-0.67	653	62	16.6	108.4	3
N-modified Cu	-0.69	400	82.3	6.8	27.2	4
[a]Cu-D	-0.68	400	64	26.8	107.2	5
Cu_2S@Cu	-0.92	400	55.8	32	128	6
multihollow Cu_2O	-0.61	355	75.2	30	106.5	7
(100)-Rich Cu	-0.67	312	90	12	37.4	8
[b]FeTPP[Cl]/Cu	-0.82	302	85	41	123.8	9
[c]N-C/Cu	-0.68	300	93.5	53.7	161.1	10
CuAg wire	-0.68	300	85.9	25.9	77.7	11
$Ce(OH)_x$/Cu	-0.7	300	80.3	43.2	129.6	12
[d]NGQ/Cu-nr	-0.9	282	80.4	52.4	147.8	13
[e]Cu-DAT	-0.69	264	68.9	30.8	81.3	14
[f]Cu_{-DS}	-0.95	100	72	67	67	15
100-cycle Cu	-0.96	68	60	27.5	18.7	16
[f]Cu_{-DS}	-1.08	30	88	71	23.5	15
Cu-Polyamine	-0.47	32	94	7	2.2	17
Low-entropy state Cu_3Sn	-1.0	6	73.7	64	3.8	18

[a]Cu-D: Cu dendrites.

[b]FeTPP[Cl]/Cu: 5,10,15,20-tetraphenyl-21H, 23H-porphine iron(iii) chloride/Cu.

[c]N-C/Cu: nitrogen-doped carbon layer on Cu.

[d]NGQ/Cu-nr: nitrogen-doped graphene quantum dots on CuO-derived Cu nanorods.

[e]Cu-DAT: Cu-3,5-diamino-1,2,4-triazole.

[f]Cu_{-DS}: Defect-site-rich Cu.

References

1. Ma WC*, et al.* Electrocatalytic reduction of CO_2 to ethylene and ethanol through hydrogen-assisted C-C coupling over fluorine-modified copper. *Nat Catal* **3**, 478-487 (2020).
2. Zhao Y*, et al.* Industrial-current-density CO_2-to-C_{2+} electroreduction by anti-swelling anion-exchange ionomer-modified oxide-derived Cu nanosheets. *J Am Chem Soc* **144**, 10446-10454 (2022).
3. Lv JJ, Jouny M, Luc W, Zhu W, Zhu JJ, Jiao F. A highly porous copper electrocatalyst for carbon dioxide reduction. *Advance Materials* **30**, e1803111 (2018).
4. Kim JY*, et al.* Quasi-graphitic carbon shell-induced Cu confinement promotes electrocatalytic CO_2 reduction toward C_{2+} products. *Nat Commun* **12**, 3765 (2021).
5. Niu ZZ*, et al.* Hierarchical copper with inherent hydrophobicity mitigates electrode flooding for high-rate CO_2 electroreduction to multicarbon products. *J Am Chem Soc* **143**, 8011-8021 (2021).
6. Zhuang T-T*, et al.* Steering post-C–C coupling selectivity enables high efficiency electroreduction of carbon dioxide to multi-carbon alcohols. *Nat Catal* **1**, 421-428 (2018).
7. Yang PP*, et al.* Protecting copper oxidation state via intermediate confinement for selective CO_2 electroreduction to C_{2+} fuels. *J Am Chem Soc* **142**, 6400-6408 (2020).
8. Wang YH*, et al.* Catalyst synthesis under CO_2 electroreduction favours faceting and promotes renewable fuels electrosynthesis. *Nat Catal* **3**, 98-106 (2020).
9. Li FW*, et al.* Cooperative CO_2-to-ethanol conversion via enriched intermediates at molecule-metal catalyst interfaces. *Nat Catal* **3**, 75-82 (2020).
10. Wang X*, et al.* Efficient electrically powered CO_2-to-ethanol via suppression of deoxygenation. *Nat Energy* **5**, 478-486 (2020).

11. Hoang TTH*, et al.* Nanoporous copper-silver alloys by additive-controlled electrodeposition for the selective electroreduction of CO_2 to ethylene and ethanol. *J Am Chem Soc* **140**, 5791-5797 (2018).

12. Luo M*, et al.* Hydroxide promotes carbon dioxide electroreduction to ethanol on copper via tuning of adsorbed hydrogen. *Nat Commun* **10**, 5814 (2019).

13. Chen C*, et al.* Highly efficient electroreduction of CO_2 to C_{2+} alcohols on heterogeneous dual active sites. *Angew Chem Int Ed* **59**, 16459-16464 (2020).

14. Hoang TTH, Ma SC, Gold JI, Kenis PJA, Gewirth AA. Nanoporous copper films by additive-controlled electrodeposition: CO_2 reduction catalysis. *ACS Catal* **7**, 3313-3321 (2017).

15. Gu Z*, et al.* Efficient electrocatalytic CO_2 reduction to C_{2+} alcohols at defect-site-rich Cu surface. *Joule* **5**, 429-440 (2021).

16. Jiang K*, et al.* Metal ion cycling of Cu foil for selective C–C coupling in electrochemical CO_2 reduction. *Nat Catal* **1**, 111-119 (2018).

17. Chen XY*, et al.* Electrochemical CO_2-to-ethylene conversion on polyamine-incorporated Cu electrodes. *Nat Catal* **4**, 20-27 (2021).

18. Shang L, Lv X, Zhong L, Li S, Zheng G. Efficient CO_2 electroreduction to ethanol by Cu_3Sn catalyst. *Small Methods* **6**, e2101334 (2022).
